# Pedestrian Positioning Using a Double-Stacked Particle Filter in Indoor Wireless Networks

**DOI:** 10.3390/s19183907

**Published:** 2019-09-10

**Authors:** Kwangjae Sung, Hyung Kyu Lee, Hwangnam Kim

**Affiliations:** 1 Development Division, Korea Institute of Atmospheric Prediction Systems, Seoul 07071, Korea; kjsung80@korea.ac.kr; 2 School of Electrical Engineering, Korea University, Seoul 02841, Korea; schk00@korea.ac.kr

**Keywords:** indoor positioning, particle filtering, dead reckoning, received signal strength (RSS) fingerprinting, sensor fusion

## Abstract

The indoor pedestrian positioning methods are affected by substantial bias and errors because of the use of cheap microelectromechanical systems (MEMS) devices (e.g., gyroscope and accelerometer) and the users’ movements. Moreover, because radio-frequency (RF) signal values are changed drastically due to multipath fading and obstruction, the performance of RF-based localization systems may deteriorate in practice. To deal with this problem, various indoor localization methods that integrate the positional information gained from received signal strength (RSS) fingerprinting scheme and the motion of the user inferred by dead reckoning (DR) approach via Bayes filters have been suggested to accomplish more accurate localization results indoors. Among the Bayes filters, while the particle filter (PF) can offer the most accurate positioning performance, it may require substantial computation time due to use of many samples (particles) for high positioning accuracy. This paper introduces a pedestrian localization scheme performed on a mobile phone that leverages the RSS fingerprint-based method, dead reckoning (DR), and improved PF called a double-stacked particle filter (DSPF) in indoor environments. As a key element of our system, the DSPF algorithm is employed to correct the position of the user by fusing noisy location data gained by the RSS fingerprinting and DR schemes. By estimating the position of the user through the proposal distribution and target distribution obtained from multiple measurements, the DSPF method can offer better localization results compared to the Kalman filtering-based methods, and it can achieve competitive localization accuracy compared with PF while offering higher computational efficiency than PF. Experimental results demonstrate that the DSPF algorithm can achieve accurate and reliable localization with higher efficiency in computational cost compared with PF in indoor environments.

## 1. Introduction

The accuracy and reliability of a localization system can greatly affect the performance for location-based applications associated with the ubiquitous and pervasive systems, including location-based services (LBS), wireless social networks, Internet-of-Things (IoT), etc. Global Navigation Satellite Systems (GNSS) such as Global Positioning System (GPS), Beidou, Global Orbiting Navigation Satellite System (GLONASS), Galileo, and Quasi-Zenith Satellite System (QZSS) are the simplest schemes for observing the positional information of a person and a vehicle outdoors. However, the availability of GNSS decreases markedly indoors, since GNSS signals may be blocked by obstacles and walls due to no line-of-sight to the satellite, noise, interference, etc. [1,2].

Hence, many indoor localization methods were designed to facilitate the positioning in indoor environments recently, and various solutions have been suggested by employing wireless infrastructures, ultrasound, motion sensing devices, and vision. The indoor positioning schemes are typically broken down into three categories: solution with wireless infrastructures (such as WiFi access points and Bluetooth transmitters), one without the infrastructures (such as dead reckoning) and sensor fusion approach using Bayes filters.

The dead reckoning (DR) scheme locates users by computing the direction and distance of movement from a known position through inertial measurement units (e.g., gyroscope and accelerometer) without the help of external references (e.g., radio infrastructures and GNSS satellites) [3,4,5]. However, inertial sensors may have large bias and lead to cumulative errors as time goes by. Furthermore, positioning inaccuracy for the DR method can be induced by random bouncing motions of the pedestrian.

A fingerprint-based method is one of the positioning approaches employing RSS values obtained by the wireless infrastructures, including cell towers [6], fixed Bluetooth modules [7,8,9], and 802.11 access points (APs) [10,11,12]. The fingerprinting approach is performed through offline and online phases. In the first phase, it collects RSS fingerprint data at positions for the localization to construct a fingerprint database (map). Then, during the second phase, the localization is executed by retrieving positions corresponding to the received RSS values via the fingerprint database. Although the site survey needed to build the fingerprint database is labor-intensive, costly, and time-consuming, since the recorded RSS values at a position are distinguished from those from other positions, the RSS data matching-based approaches have been widely employed for indoor localization. Nonetheless, since RF signals are unpredictable and time-varying due to obstacles and multipath fading, the RSS fingerprinting schemes in practice cannot offer satisfactory positioning results for the user. For example, Molina et al. [13] show that the Google geolocation API that determines indoor location via information about cell towers and WiFi APs in Google Maps can provide the average accuracy of less than 30 m in 50% for indoor environments with many obstructions.

For the localization system, Bayesian filters are used to combine sensory data form disparate sources to obtain better positioning accuracy. The Kalman filter (KF) and extended Kalman filter (EKF) as suboptimal Bayesian algorithms have been applied to indoor pedestrian navigation [14]. However, they cannot be used for the navigation system with severe nonlinearity and non-Gaussian noises [15]. On the contrary, the unscented Kalman filter (UKF) is founded on the intuition that it is easier to approximate a probability distribution than to approximate a nonlinear equation [16]. The UKF, however, cannot apply to the nonlinear system models with highly non-Gaussian noise [17]. The particle filtering (PF) can approximate the true posterior distribution of the state for the nonlinear/non-Gaussian system [17,18].

The iBeacon, a novel type of Bluetooth module introduced by Apple, provides mobile devices (such as smartphone) with positional data using the Bluetooth 4.0 technology, also known as Bluetooth low-energy (BLE) [19]. This Bluetooth module can be used to improve existing methods and to develop more reliable and accurate indoor localization algorithms [20,21].

Among the filtering algorithms, the particle filtering algorithm offers the most accurate positioning results, and thus, it has been widely used for the indoor positioning. Various positioning methods that fuse positional information from the RSS fingerprint-based method and DR technique on smartphones using PF have been suggested to provide more accurate indoor positioning information [22,23,24]. In general, they predict the pedestrian position using the movement and position information of the pedestrian obtained by inertial measurement units (IMUs), and then the predicted position is updated the estimated position using location data determined by the measured RSS values via the fingerprint map. GIFT [25] uses a more stable gradient for RF signal data rather than the signal value to address biased and time-variant WiFi signal readings across sensing devices as well as changes in transmission power of WiFi APs. By comparing RSS values at neighboring positions, GIFT generates a fingerprint map based on RSS gradients called Gmap. Then, it estimates the position of users by integrating the RSS measurements and motion detection information using an extended particle filter; that is, their locations are predicted through mobility sensing results and are updated based on the comparison between Gmap and measured RSS values. SLAC [11] is a fingerprint localization algorithm that performs system calibration and indoor positioning simultaneously, where user step counter readings and WiFi RSS fingerprints are jointly considered using a specialized particle filter. It learns parameters of step length model, calibrates RF signal data owing to heterogeneous devices, and meanwhile estimate the user location accurately by solving a convex optimization problem. In this fusion algorithm, the user location is predicted through walking displacements obtained by the step length model and is updated by the convex optimization localization and specialized particle filter. However, due to high computational cost required to solve the convex optimization problem, the smartphone-based localization system using SLAC may not be applied to real-time positioning applications. Furthermore, a large sample (particle) size of PF required for better localization accuracy as in [22,24,25] can result in a substantial computation time when comparing to Kalman filtering-based methods. Table 1 summarizes the main technique, experimental environment, sample size, localization accuracy of the aforementioned PF-based localization methods along with the proposed algorithm in this paper.

In our previous work, the capability of estimating the user’s position by fusing DR and RSS fingerprinting approaches via the improved Kalman filtering agorithm, denoted the sigma-point Kalman particle filter (SKPF) has already been addressed [26]. By utilizing the sample weighting scheme used in the PF and the deterministic sampling approach of the UKF, the SKPF method can provide more accurate localization results compared with UKF and KF. Our investigations are now updated with improvements in the fusion process of DR and fingerprinting method using an enhanced PF for the indoor positioning. The contributions of this study are as follows:

- We design a sample weighting method that calculates a weight for each particle through the likelihood function of positional measurements based on kernel density estimation. Similar to the SKPF [26], the enhanced PF scheme predicts the location of the user for every user step using the mobility sensing information determined by the gyroscope and accelerometer. Then, like SKPF, it also corrects the predicted position through the user’s positional observations obtained from the fingerprinting approach based on machine learning that uses WiFi and iBeacon RSS values as location features. The SKPF evaluates the weight of particles obtained from the deterministic sampling of the UKF through the likelihood function based on parametric technique (e.g., Gaussian function) as in general PF, such as sequential importance resampling (SIR) filter [18]. On the contrary, the enhanced PF computes the weight of particles drawn by the importance sampling [27] through the likelihood function calculated by Gaussian kernel density estimation (i.e., Parzen-window method) among nonparametric techniques [28].
- For the enhanced PF, the likelihood of positional measurements is represented by the target distribution, which is generated based on point mass representations using positional measurements obtained from the measured WiFi and iBeacon RSS values and pedestrian direction data using the fingerprinting algorithm. The RSS data received from WiFi and iBeacon APs permits the target distribution to reflect indoor wireless environments surrounding the user. Unlike the localization schemes [11,22,23,24,25,26] shown in Table 1 that calculate the weight of samples through the parametric density estimation, the particle weight in the enhanced PF is determined by calculating a probability density function of the target distribution using the Parzen-window density estimation for better positioning results.
- We propose a double-stacked particle filter (DSPF) as the improved PF. The DSPF estimates the location of the pedestrian using a separate particle presentation for both of the proposal and target distributions. Using the target distribution that reflects wireless circumstances surrounding the user through the multiple observations, the DSPF can conduct reliable position estimation in indoor wireless environments affected by considerable bias and errors. Also, the DSPF can perform accurate position estimations even with less particles due to the use of target distribution. Furthermore, the use of a small particle size guarantees a reduction in the computational cost and makes it possible for the DSPF to be applied to real-time localization applications.
- We have implemented the DSPF-based localization system on smartphones, and performed indoor positioning experiments in a campus bulling. Experimental results indicate that the DSPF can offer more accurate localization performance compared with the UKF and KF, and can achieve localization results that are as accurate as PF, while it provides better computational efficiency than PF.

This paper is organized in the following manner. Section 2 demonstrates the overall positioning system configuration. Components of the indoor positioning system are discussed in more detail in Section 3. Section 4 describes the experimental environment used for the performance analysis, and Section 5 provides results from pedestrian localization experiments in the indoor environments and compares DSPF with PF, UKF, and KF. Finally, Section 6 summarizes the localization performance for the DSPF algorithm.

## 2. System Configuration

**Figure 1:**
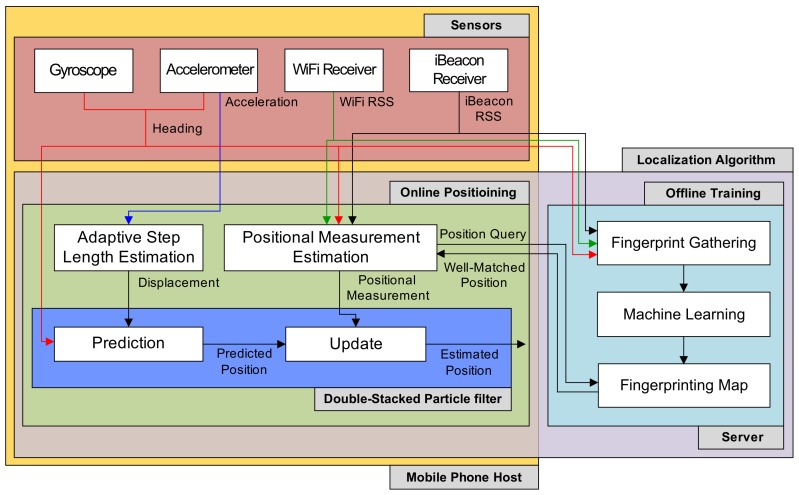
Localization system architecture.

Figure 1 indicates the positioning system used in this study, which is operated on a web server and a mobile phone client. The mobile phone is employed to obtain the motion and position information of the pedestrian from its IMU sensors and then to locate the pedestrian using the data. The machine learning algorithm for localizing the user is carried out on the server. Also, the server is used to process positional query data received from the phone via web. The position estimation system is composed of localization schemes and sensors.

The sensors include motion sensing devices and radio modules in the phone. The radio modules consist of iBeacon and WiFi receivers, and they offer RSS values obtained from iBeacon transmitters and WiFi APs for localization algorithms. The motion sensing devices contain the gyroscope used to obtain the orientation and angular velocity and the acceleration gauge used to determine a three-axis accelerations. Sensing information obtained from IMU devices (gyroscope and accelerometer) and radio modules are employed for position estimate in the localization schemes, including the DSPF algorithm and machine learning.

The localization methods are performed by online positioning and offline training phases. In the training step, RSS values received by the radio modules are recorded at chosen positions through the mobile phone. Moreover, pedestrian direction information obtained from the IMU sensors are also collected, since the body of the user similar to the obstacle can have a great impact on the RF signals between the radio transmitters and receivers. Then, the recorded information are transmitted into web server and are converted into fingerprinting database (map) using the machine learning algorithm.

During the positioning phase, the displacement calculation, positional measurement inference, and DSPF are executed for estimating the position of the pedestrian. DSPF is performed through dead reckoning and update (correction) steps, in which pedestrian locations are inferred using a user motion model described in Section 3.3.

During the prediction phase, referred to as dead reckoning (DR), the location of the user for every step of the user is predicted using the movement direction information determined by the gyroscope and accelerometer and the user’s displacement calculated from measurements of the accelerometer. A more detailed description for both the direction determination and displacement (step length) calculation is addressed in Section 3.1.

In the correction step, the pedestrian position obtained from the prediction phase is corrected using the positional measurement for the user, which is gained from external observers, such as GNSS. However, because of the GNSS’s unavailability indoors, observations employed in the update phase are gained from the fingerprint database constructed by the machine learning algorithm in the offline training step. When position queries with pedestrian heading data and measured RF signals are sent by the mobile phone into the web server side, the optimal pedestrian position that matches query data (RSS readings and heading angle) is estimated through fingerprinting map. Then, the machine learning algorithm transmits the estimated location of the pedestrian back to the mobile phone. More details on the machine learning approach for estimating the positional measurement are explained in Section 3.2. The DSPF algorithm based on the two-phase process (prediction and update) is discussed further in Section 3.4.

## 3. Localization Algorithm

The heading angle determination of the user, estimation for step length and positional measurement, user motion model, and DSPF method that are required for performing our positioning algorithm are explained in the following subsections.

### 3.1. Step Length Estimation and Heading Determination

During the user’s movements, the readings of the three-axis accelerometer have the evident periodicity for each of its axes compared with the state of the static user. Compared to when the user remains stationary, the change in accelerometer readings while the user walks from place to place exhibits a periodic pattern. Therefore, the accelerometer readings have been employed for user step detection and stride length measurement by analyzing the acceleration magnitudes and patterns [29]. The displacement (step length) estimation method designed in [26] demonstrates the relationship of a maximum acceleration value measured by the smartphone and displacement of the user as follows:

(1) $$d=c_{1}p^{2}+c_{2}p+c_{3}$$

where the values of *p* and *d* denote a peak value of the acceleration measurements obtained by the three-axis accelerometer and the estimated displacement, respectively. Based on experimental results in [26], $c_{1}$, $c_{2}$, and $c_{3}$ are set to −412.93, 416.24, and 5.71, respectively.

The heading angle of the user is essential to provide better localization results indoors [30]. Various studies have been proposed to estimate an accurate heading direction of the pedestrian. Many of the works fuses measurements from gyroscope and digital compass on smartphone using the KF to obtain optimized direction information [31,32,33]. For some works, Principal Component Analysis (PCA) is used to infer an accurate pedestrian heading through sensing information measured by gyroscope and accelerometer [34,35,36]. Kang and Han [37] and Kang et al. [38] integrate measurements from IMU sensors based on weighted models. Furthermore, Xiao and Wen [39] uses least-squares linear regression to estimate an optimal pedestrian heading through accelerometer readings.

For our localization system, the direction data is obtained from an iOS Core Motion framework [40] as in [26]. The information measured by built-in IMU sensors (such as gyroscope and accelerometer) in the iPhone can offer a reliable heading data for the LBS applications implemented on the iPhone; however, if the user performs our positioning system on an Android phone, the heading information is obtained from Android libraries with regard to the motion sensing [41]. The estimated displacement and direction measurement are used in the prediction (DR) step of the DSPF algorithm.

### 3.2. Inference of Positional Measurement

For the indoor environments, the GNSS signal does not ensure that the pedestrian position is determined accurately because of its unavailability indoors. To localize a pedestrian indoors, RSS fingerprints have been widely used in the localization approaches, because the radio frequency RSS values can be easily obtained using off-the-shelf products, including the Bluetooth and WiFi modules.

Using the RSS data, the machine learning approach in our indoor positioning system can estimate pedestrian positions instead of GNSS. Based on experimental results in [26], we used NBC (naive Bayes classifier) as a machine learning algorithm in this study, since it can yield better positioning results than other conventional methods, including SVM (support vector machine), KNN (k-nearest neighbors algorithm), and ANN (artificial neural network). For further details on its implementation, see [26]. The process to determine the user’s location with the machine learning method is composed of online and offline steps.

During the offline step, the pedestrian direction data mentioned in Section 3.1 along with the RSS values measured from the iBeacon transmitters and 802.11 access points are recorded using the smartphone of the user at the positions predetermined for the localization indoors, similarly as in Wi-Fi fingerprint-based approaches that uses the heading direction to improve the RSS-to-position mapping [10,42,43]. Then, the recorded fingerprint data as training information are transmitted to the server and are converted into fingerprinting map by the machine learning method.

In the online step, position queries that include the measured direction data and RSS values are transmitted into the web server by the mobile phone. Then, the best matched location of the user with the query data is inferred using the fingerprinting map generated by the machine learning algorithm and is sent back into the mobile phone. Instead of GNSS, the estimated position from the machine learning scheme is employed as an observation for pedestrian position in the DSPF method.

### 3.3. Pedestrian Model

**Figure 2:**
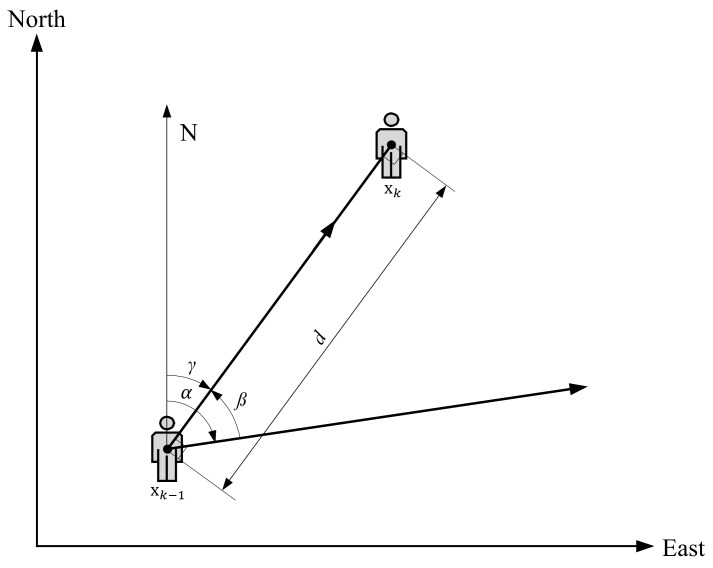
User motion model that takes into account the user’s *x* (east) and *y* (north) coordinates and heading angles at timesteps *k* and k−1.

The reduced scheme of the pedestrian model used in our positioning approach is shown in Figure 2. The user’s *x*-axis and *y*-axis coordinates in indoor space at timestep *k* can be written as state vector $x_{k}={\left[\begin{array}{cc} x_{k} & y_{k} \end{array}\right]}^{T}$. For the pedestrian model, direction information at times *k* and k−1 are indicated by the angles γ and α respectively, and a counterclockwise angle between state vector $x_{k-1}$ and $x_{k}$ is represented by the value of β. The user’s displacement (step length) between times k−1 and *k* is denoted by the value of *d*.

For the pedestrian model that indicates the user movement using IMU sensors, user location $x_{k}$ can be determined as follows:

(2) $$\begin{array}{ccc} x_{k} & = & x_{k-1}-d\cos\left(\alpha \right)\sin\left(\beta \right)+d\sin\left(\alpha \right)\cos\left(\beta \right) \end{array}$$

(3) $$\begin{array}{ccc} y_{k} & = & y_{k-1}+d\cos\left(\alpha \right)\cos\left(\beta \right)+d\sin\left(\alpha \right)\sin\left(\beta \right) \end{array}$$

where α and β are obtained from a gyroscope and accelerometer, and *d* is obtained from a peak value of the measured acceleration data between times k−1 and *k*, as explained in Section 3.1. Also, Equations (2) and (3) where the sine and cosine rule is applied and α−β is replaced with γ can be written as

(4) $$\begin{array}{ccc} x_{k} & = & x_{k-1}+d\sin\left(\gamma \right) \end{array}$$

(5) $$\begin{array}{ccc} y_{k} & = & y_{k-1}+d\cos\left(\gamma \right). \end{array}$$

Assuming that the process noise $w_{k-1}$ and observation noise $v_{k}$ are uncorrelated with zero-mean and covariances $Q_{k-1}$ and $R_{k}$ respectively, state transition and observation models for the user movement are represented as follows:

(6) $$\begin{array}{ccc} x_{k} & \;\;\;\;=\;\;\;\; & F_{k-1}x_{k-1}+G_{k-1}d+w_{k-1} \end{array}$$

(7) $$\begin{array}{ccc} z_{k} & \;\;\;\;=\;\;\;\; & H_{k}x_{k}+v_{k} \end{array}$$

where $F_{k-1}$ is identity matrix and $G_{k-1}={\left[\begin{array}{cc} \sin(\gamma ) & \cos(\gamma ) \end{array}\right]}^{T}$. In Equation (7), the value of $z_{k}$ indicates the observation ${\left[\begin{array}{cc} x_{k} & y_{k} \end{array}\right]}^{T}$ for the user position, which is determined by the machine learning algorithm, and $H_{k}$ is identity matrix, because the observation space is the same as the model state space. The observation noise $v_{k}$ results from the measurement estimation inaccuracy of the machine learning scheme.

### 3.4. Double-Stacked Particle Filter (DSPF)

In this section, we describe the principle of PF used to devise the DSPF algorithm (Section 3.4.1), the DSPF algorithm for the pedestrian positioning (Section 3.4.2), and our localization method based on the DSPF algorithm (Section 3.4.3).

#### 3.4.1. Particle Filter

This subsection introduces the most basic sequential PF that estimates recursively the model state using observations. For PF, $N_{s}$ samples $\left\{x_{0:k}^{i},i\;=\;1,\ldots ,N_{s}\right\}$ approximates the posterior density as follows:

(8) $${\left\{x_{0:k}^{i},w_{k}^{i}\right\}}_{i=1}^{N_{s}}∼p\;\left(x_{0:k}|z_{1:k}\right)$$

where $x_{0:k}\;=\;\left\{x_{j},j\;=\;0,\ldots ,k\right\}$ indicates entire system states up to timestep *k*, and $\left\{w_{k}^{i},i\;=\;1,\ldots ,N_{s}\right\}$ denotes the sample’s weight such that $\sum_{i}w_{k}^{i}\;=\;1$. Based on the importance sampling method [27], the each sample’s weight can be determined by

(9) $$\begin{array}{ccc} w_{k}^{i} & \propto & \frac{target\;distribution}{proposal\;distribution}=\frac{p\;\left(x_{0:k}^{i}|z_{1:k}\right)}{q\;\left(x_{0:k}^{i}|z_{1:k}\right)}\\ = & w_{k-1}^{i}\frac{p\;\left(z_{k}|x_{k}^{i}\right)p\;\left(x_{k}^{i}|x_{k-1}^{i}\right)}{q\;\left(x_{k}^{i}|x_{k-1}^{i},z_{1:k}\right)}. \end{array}$$

By using the sample weight and delta function $\delta \;\left(\cdot \right)$ centered on each sample, the posterior density can be also constructed as:

(10) $$p\;\left(x_{0:k}|z_{1:k}\right)\approx \sum_{i=1}^{N_{s}}w_{k}^{i}\delta \;\left(x_{0:k}-x_{0:k}^{i}\right)$$

Note that we employ only a filtered posterior density, since DSPF is based on the sequential importance resampling (SIR) algorithm [27]. Thus

(11) $$p\;\left(x_{k}|z_{1:k}\right)\approx \sum_{i=1}^{N_{s}}w_{k}^{i}\delta \;\left(x_{k}-x_{k}^{i}\right).$$

#### 3.4.2. Double-Stacked Particle Filter for Pedestrian Localization

**Figure 3:**
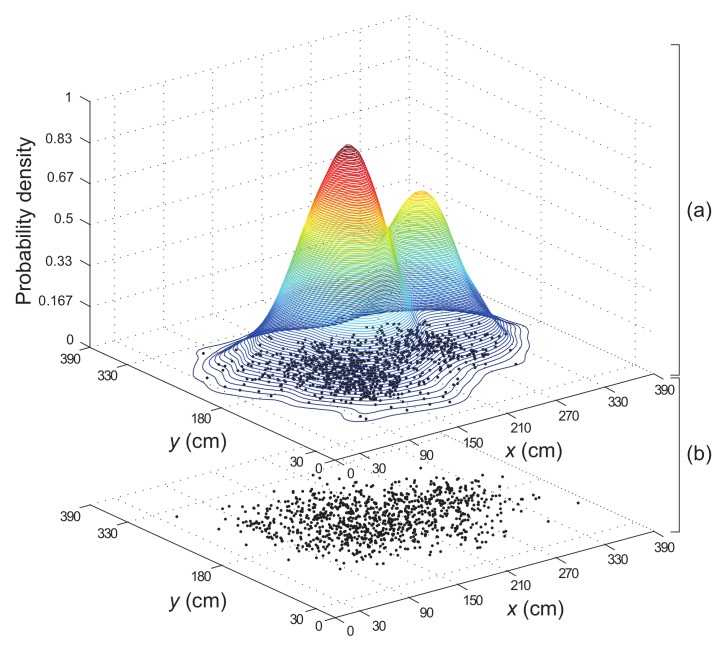
DSPF algorithm with (**a**) target and (**b**) proposal distributions that consist of particles with *x*-axis and *y*-axis coordinate values.

The DSPF algorithm proposed in this paper is comprised of the target and proposal distributions based on point mass representations with their own sample (particle) size (i.e., $N_{pdp}$ samples for proposal distribution, which means particles used in general PFs (e.g., $N_{s}$ samples in Section 3.4.1), and $N_{tdp}$ samples for target distribution), as shown in Figure 3. It should be noted that the sample sizes $N_{pdp}$ and $N_{tdp}$ are determined based on some sensitivity experiments so that the estimated state by the DSPF can approximate the true state. With the two distributions, the DSPF algorithm is executed through two phases: a prediction and an update.

##### (1) Prediction

During this step, the model state $x_{k}$ at timestep *k* can be predicted through importance sampling of the particles ${\left\{x_{k-1}^{i}\right\}}_{i=1}^{N_{pdp}}$ for proposal distribution. The estimate accuracy of PF is most affected by the importance sampling. As a consequence, it is required to design the proposal distribution that can approximate the posterior distribution well. However, since it is difficult to construct such a proposal distribution, sampling from the probabilistic model of the state transition (prior distribution) is commonly employed as the proposal distribution [44]. That is, similar to the SIR filter, the importance density (i.e., proposal distribution) for the DSPF algorithm is chosen to be the prior density as follows:

(12) $$q\;\left(x_{k}|x_{k-1}^{i},z_{1:k}\right)=p\;\left(x_{k}|x_{k-1}^{i}\right).$$

Therefore, the samples ${\left\{x_{k}^{i}\right\}}_{i=1}^{N_{pdp}}$ for the proposal distribution in the importance sampling stage of the DSPF algorithm at timestep *k* are drawn from the prior distribution $p\;\left(x_{k}|x_{k-1}^{i}\right)$ given by the state transition model (e.g., Equation (6)).

##### (2) Update

For this step, the propagated state using the state transition model in the prediction phase can be corrected using the measurements observed for the model state at timestep *k*. By applying the prior density (12) to Equation (9), the likelihood function $p\;\left(z_{k}|x_{k}^{i}\right)$ of observations denotes the target distribution, and it can be used to compute the weight of the particle in the proposal distribution. Therefore

(13) $$w_{k}^{i}\propto w_{k-1}^{i}p\;\left(z_{k}|x_{k}^{i}\right).$$

To proceed with the target distribution estimate, we assume that observations $z_{k}$ have a great impact on the estimate of the model state $x_{k}$, which means the user’s positional information ${\left[\begin{array}{cc} x_{k} & y_{k} \end{array}\right]}^{T}$ in this study. This is a reasonable assumption given that the posterior density is proportionate to the likelihood of the observations $p(z_{k}^{1},\ldots ,z_{k}^{N_{o}}|x_{k})$, multiplied by the prior density $p(x_{k})$, and divided by the normalizing constant $p(z_{k}^{1},\ldots ,z_{k}^{N_{o}})$ as follows:

(14) $$p\left(x_{k}|z_{k}^{1},\ldots ,z_{k}^{N_{o}}\right)=\frac{p\left(z_{k}^{1},\ldots ,z_{k}^{N_{o}}|x_{k}\right)p\left(x_{k}\right)}{p(z_{k}^{1},\ldots ,z_{k}^{N_{o}})}$$

where ${\left\{z_{k}^{i}\right\}}_{i=1}^{N_{o}}$ is a set of $N_{o}$ observations at time *k*. Therefore, the observations from external observers can be used to estimate the target distribution, since the target distribution can be given by the likelihood as mentioned above.

As can be noticed in Algorithm 1, the DSPF algorithm constructs the target distribution $p(z_{k}^{1},\ldots ,z_{k}^{N_{o}}|x_{k})$ that is composed of normally distributed random samples ${\left\{x_{k}^{j}\right\}}_{j=1}^{N_{so}}$ with the number of samples $N_{so}$ and standard deviation $\sigma_{z_{k}^{i}}$ for the each observation $z_{k}^{i}$, where $N_{so}$ is calculated by dividing the number of samples for the target distribution $N_{tdp}$ by $N_{o}$, and $\sigma_{z_{k}^{i}}$ can be obtained experimentally by trial and error in such a way that the estimated state by the DSPF would not drift too far from the ground truth. The choice of $\sigma_{z_{k}^{i}}$ for our localization method based on the DSPF is explained in Section 3.4.3. The use of observations permits the target distribution to reflect indoor wireless environments surrounding the user.

**Algorithm 1** Estimation of Target Distribution.
1:Input $N_{tdp},N_{o},z_{k}^{i},\sigma_{z_{k}^{i}}$                                        ▹ constants2:$N_{so}\leftarrow N_{tdp}/N_{o}$                                                ▹sample size for each observation3:**for**$i=1:N_{o}$                                                       ▹ sampling4:   
**for** $j=1:N_{so}$
5:     
$x_{k}^{j}\leftarrow z_{k}^{i}+N\left(0,\sigma_{z_{k}^{i}}^{2}\right)$
6:   
**end for**
7:
**end for**



The target distribution (i.e., likelihood function $p\;\left(z_{k}|x_{k}^{i}\right)$) is calculated using nonparametric density estimation to estimate a probability density function with unknown parameters (details of the nonparametric technique can be found in [28]). Thus, using Gaussian kernel density estimation via the Parzen-window method among the nonparametric techniques, the probability density function (PDF) for the target distribution is calculated by

(15) $$p\left(z_{k}|x_{k}^{i}\right)=\frac{1}{N_{tdp}h_{x}h_{y}}\;\sum_{j=1}^{N_{tdp}}\;\frac{1}{2\pi }\exp\;\left(\;\;-\frac{1}{2}\;\left[\;{\left(\frac{x_{k}^{i}-x_{k}^{j}}{h_{x}}\right)}^{2}\;+\;{\left(\frac{y_{k}^{i}-y_{k}^{j}}{h_{y}}\right)}^{2}\;\right]\;\right)$$

where $\left(x_{k}^{i},y_{k}^{i}\right)$ indicates *x* and *y* coordinates for each sample $x_{k}^{i}$ with regard to proposal distribution represented by Figure 3b, $\left(x_{k}^{j},y_{k}^{j}\right)$ represents *x*-axis and *y*-axis position of each sample $x_{k}^{j}$ for target distribution illustrated by Figure 3a. The kernel (bandwidth) sizes $h_{x}$ and $h_{y}$ are parameters that have a significant impact on the density estimation. For this study, these values are determined by the optimal bandwidth selection method of [45] as follows:

(16) $$\begin{array}{ccc} h_{x} & \;\;\;\;=\;\;\;\; & {\hat{\sigma }}_{x}{\left(\frac{1}{N_{tdp}}\right)}^{\frac{1}{6}} \end{array}$$

(17) $$\begin{array}{ccc} h_{y} & \;\;\;\;=\;\;\;\; & {\hat{\sigma }}_{y}{\left(\frac{1}{N_{tdp}}\right)}^{\frac{1}{6}} \end{array}$$

where ${\hat{\sigma }}_{x}$ and ${\hat{\sigma }}_{y}$ are standard deviations for *x*-axis position data ${\left\{x_{k}^{j}\right\}}_{j=1}^{N_{tdp}}$ and *y*-axis position data ${\left\{y_{k}^{j}\right\}}_{j=1}^{N_{tdp}}$ of samples in the target distribution estimated by Algorithm 1, respectively.

We can substitute Equation (15) into Equation (13) to provide the means of calculating the weight $w_{k}^{i}$ of each sample $x_{k}^{i}$ for the proposal distribution. The resulting weight $w_{k}^{i}$ can be expressed as

(18) $$w_{k}^{i}\propto w_{k-1}^{i}\frac{1}{N_{tdp}h_{x}h_{y}}\;\sum_{j=1}^{N_{tdp}}\;\frac{1}{2\pi }\exp\;\left(\;\;-\frac{1}{2}\;\left[\;{\left(\frac{x_{k}^{i}-x_{k}^{j}}{h_{x}}\right)}^{2}\;+\;{\left(\frac{y_{k}^{i}-y_{k}^{j}}{h_{y}}\right)}^{2}\;\right]\;\right).$$

After normalizing the weights, the estimated state ${\bar{x}}_{k}$ by the weighted sum method is given by

(19) $${\bar{x}}_{k}=\sum_{i=1}^{N_{pdp}}x_{k}^{i}w_{k}^{i}.$$

The common issue with PF is the filter degeneracy problem, where all but one sample have very small weights after a few iterations [18]. The effective sample size ${\hat{N}}_{\mathrm{eff}}$ as a value that indicates the magnitude of the degeneracy problem can be written as

(20) $${\hat{N}}_{\mathrm{eff}}=1/\sum_{i=1}^{N_{pdp}}{\left(w_{k}^{i}\right)}^{2}.$$

That ${\hat{N}}_{\mathrm{eff}}$ falls below the sample size threshold $N_{T}$ implies severe degeneracy. To mitigate the effects of the degeneracy, the resampling is applied whenever the degeneracy problem is detected (i.e., ${\hat{N}}_{\mathrm{eff}}\leq N_{T}$). The resampling stage aims to remove the samples with a small value of weight and to focus on the samples that have a large value of weight. The resampling step of the DSPF algorithm is implemented with the systematic resampling [46], because it minimizes the variance of importance weights and is simple to implement.

#### 3.4.3. DSPF-Based Positioning Algorithm

A pseudo-code explanation of our localization algorithm based on the DSPF can be summarized as Algorithm 2. For our positioning system, the pedestrian movement at timestep *k* is expressed as state vector with positional information $x_{k}={\left[\begin{array}{cc} x_{k} & y_{k} \end{array}\right]}^{T}$ via the mobility model as described in Section 3.3. Hence, for the dead reckoning (prediction) phase, the pedestrian state $x_{k}$ is predicted through the particles propagated by the state transition model (6) that leverages the user stride step and heading direction measured IMU sensors; in other word, the proposal distribution for the state $x_{k}$ is generated using Equation (6).

During the update step, the predicted user position is corrected using the target distribution estimated by Algorithm 1 through the positional measurements obtained from the machine learning algorithm introduced in Section 3.2. Sensitivity experiments (not reported here) showed that the choice of standard deviation $\sigma_{z_{k}}=8.36$ for the measurements in Algorithm 1 results in a high correlation between the ground truth and the estimated state by the DSPF for this study; however different choices of the values can be required for more complex estimation problems. The weights of particles for the proposal distribution are evaluated by calculating a probability density function of the target distribution using the Parzen-window density estimation (see Equation (18)). After normalizing the particle weights, the updated (corrected) position ${\bar{x}}_{k}$ is obtained by the weighted sum using Equation (19). Then, particles are resampled to achieve better filter performance.

By estimating the target distribution that reflects indoor wireless environments surrounding the user through multiple observations, the DSPF method can provide more accurate positioning results compared with UKF and KF, achieving localization accuracy comparable with PF while providing higher computational efficiency than PF. The positioning accuracy and computational performance for the DSPF algorithm are discussed further in Section 5.1 and Section 5.3, respectively. However, due to the proposal and target distributions based on point mass representations, the use of excessive particles in the DSPF algorithm may lead to the considerable computation time. Hence, the particle size need to be properly selected depending on indoor wireless network circumstances. The selection of the sample size is discussed further in Section 5.2.

**Algorithm 2** DSPF-Based Positioning Algorithm.

$\left[{\left\{x_{k}^{j},w_{k}^{j}\right\}}_{j=1}^{N_{pdp}}\right]=\mathrm{DSPF}\left[{\left\{x_{k-1}^{j},w_{k-1}^{j}\right\}}_{j=1}^{N_{pdp}},z_{k}\right]$

1:Input $N_{pdp}$            ▹ constants2:**for**$j=1:N_{pdp}$            ▹ generate proposal distribution3:   Sample  $x_{k}^{j}∼p\left(x_{k}|x_{k-1}^{j}\right)$ using Equation (6)4:
**end for**
5:Estimate target distribution using Algorithm 16:**for** $j=1:N_{pdp}$            ▹ compute weight of each sample7:   Calculate weight using Equation (18)8:
**end for**
9:Determine the sum of sample weights: $t\;=\;\sum_{j}w_{k}^{j}$10:**for** $j=1:N_{pdp}$            ▹ normalize weights11:   Normalize:  $w_{k}^{j}=t^{-1}w_{k}^{j}$12:
**end for**
13:Estimate the current position ${\bar{x}}_{k}$ using Equation (19)14:Compute the effective number of samples from Equation (20)15:**if** ${\hat{N}}_{\mathrm{eff}}\leq N_{T}$            ▹ resample16:   Resample particles through the systematic method [46]17:
**end if**



## 4. Experiment Setup

**Figure 4:**
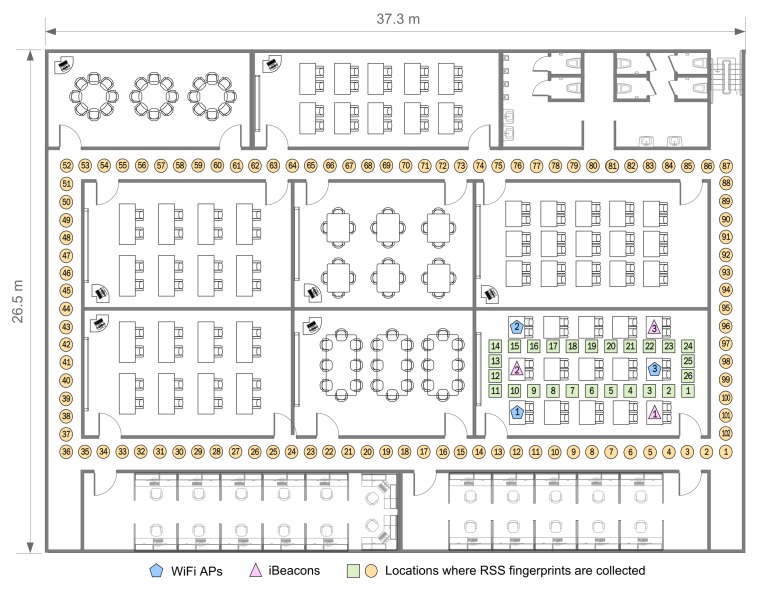
Floor map for experimental testbeds. RSS fingerprints and user’s direction information are collected in both the hallways and room.

Several localization experiments are constructed to execute the fingerprinting method based on the machine learning and to compare the pedestrian positioning accuracy for the filters indoors, such as UKF, KF, DSPF, and PF. Our localization system is performed on a web server and a smartphone client (iPhone5S). The server is employed to provide the positional measurements used in the DSPF algorithm using the fingerprinting scheme. The smartphone is used to localize the user through the DSPF algorithm that fuses noisy user motion information obtained from the DR and positional measurements obtained from the server. A detailed description of our positioning system can be found in Section 2. The indoor test site for our experiments is indicated in Figure 4. The test site has the dimensions of about 37.3 by 26.5 m. In this study, we aim to examine the feasibility and accuracy of the DSPF as a positioning algorithm in indoor environments. Hence, our experiments were performed in a corridor and a room in a building that are often used to verify the performance of the positioning algorithms as shown in Table 1 rather than a complex environment; however, we need to examine the positioning performance of the DSPF for pedestrians that move from place to place in more complex indoor environments in the future, including the airport (large open space), shopping mall, and library.

Our experiments were conducted using iBeacons (Estimote) and WiFi APs (ipTime N104T), whose locations are denoted by blue pentagons and pink triangles marked as shown in this Figure 4, respectively. The wireless network composed of both WiFi APs and iBeacons works on 2.4 GHz band. Each WiFi AP was equipped with a wireless adapter that provide a high-speed data transmission of up to 150 Mbps using IEEE 802.11n. The iBeacon that needs a maximum 10 mW transmission power via the Bluetooth Low Energy (BLE) technology offers a maximum 100 m wireless coverage and a data transfer speed of up to one Mbps. We used iPhone5S as a mobile host to record RSS fingerprints from iBeacons and WiFi APs and to localize the pedestrian, which operates with IMU sensors (such as gyroscope and accelerometer) as well as Bluetooth 4.0 and WiFi 802.11n adapters. The WiFi RSS information on iPhone5s was obtained from private Apple80211 framework [47]. This is because public APIs for scanning RSS values of Wi-Fi networks are not provided by Apple. For the smartphone, the measurements of the gyroscope and accelerometer is updated every 10 ms, and the sampling frequency of iBeacon and WiFi receivers is one Hz.

In our experiments, 50 pedestrians of age range from 20 to 40 participated, striding at various speeds. As can be seen in Figure 4, the positions where fingerprint information composed of heading angle data and RSS values are collected by the mobile host of the user are schematically represented by orange circles and green squares. They are located at intervals of one meter with corresponding sequence number to each physical location. We recorded over 100 location data at every physical position to build the fingerprint map.

Owing to the obstacles between RF receivers and transmitters, RSS information received from one radio transmitter may change remarkably for different positions. Therefore, we intentionally deployed all radio transmitters (iBeacons and WiFi APs) in the same place (i.e., the lecture room) in order to analyze the effect of the obstructions (such as walls) on RSS values in indoor environments. Considering this deployment, our experiments are divided into two test cases for the performance analysis of our system: TC1 and TC2.

For TC1, the user with the smartphone strides on the positions of the green squares clockwise. This case reflects the good wireless environments between the RF transmitters and receivers (mobile host), because there are no obstacles. On the other hand, for TC2, the pedestrian strides on the locations of orange circles clockwise. This test case indicates the poor radio environments in which RF signals are blocked because of walls between RF transmitters and receivers. For TC1 and TC2, the ground truth information were obtained by measuring their *x*-axis and *y*-axis coordinates using a measuring tape while walking along the physical locations (green squares and orange circles in Figure 4).

**Figure 5:**
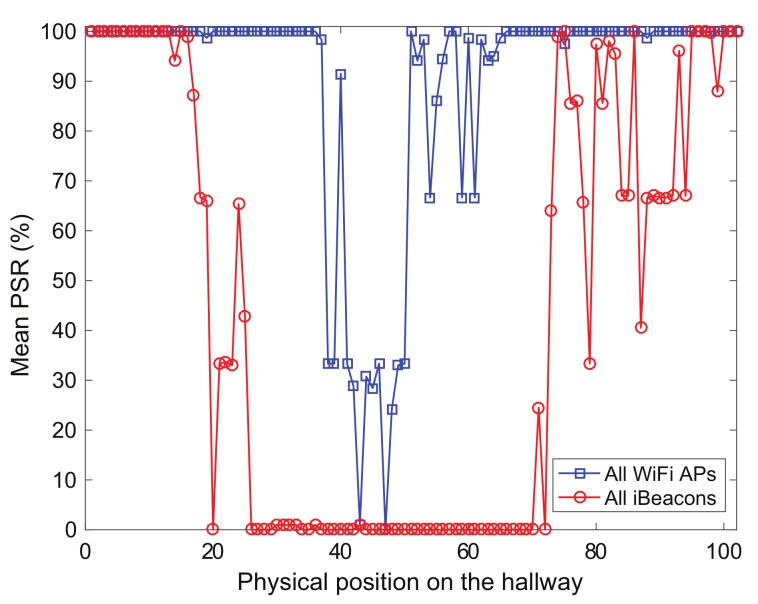
Mean packet success rate (PSR) computed using pedestrians’ smartphones in test positions (orange circles in Figure 4) of test case TC2.

The mean packet success rate computed using RF signals from the transmitters in the hallway (orange circles) of TC2 is shown in Figure 5. The RF signals from the transmitters can be used to compute the packet success rate corresponding to each RF transmitter. The mean packet success rate can provide a reasonable indication of how the wireless environment is good at a given location. For example, the farther the pedestrians walk on the physical locations from the RF transmitters, the more the packet success rate decreases, as shown in Figure 5 that indicates the mean packet success rate obtained from pedestrians’ smartphones for each test position of TC2. The mean packet success rate values for the iBeacons and WiFi APs at all physical locations for TC2 are approximately 44% and 90% respectively, while those for TC1 are about 100%, together. Note that the positioning systems in this experiment use only the RSS values obtained from the RF transmitters to locate the user, but do not require to receive communication packets from them.

## 5. Indoor Localization Experiments

This section presents the results of the performance evaluation for our positioning approach in real environments.

### 5.1. Positioning Accuracy

To investigate the performance of the localization method proposed in this study, several experimental tests are executed in indoor environments. For the performance analysis, 50 pedestrians with the mobile phone on which our localization system is implemented strode on positions marked with orange circles and green squares clockwise in test cases TC1 and TC2 and applies the algorithm to estimate his/her own position.

The features and notations of localization algorithms employed in this tests are summarized by Table 2. The DR approach predicts the pedestrian’s location through the motion information (direction and acceleration) measured from the IMU sensors on the smartphone. For the localization methods, the observations for the pedestrian location are determined by the fingerprinting approach based on NBC instead of GNSS, as explained in Section 3.2. The position data gained from the fingerprinting and DR methods can have the localization errors. The DSPF method is employed to achieve better localization performance by combining noisy location information determined by the fingerprinting and DR approaches. The DSPF scheme depending on the type of the training information employed in the fingerprinting scheme that uses the machine learning algorithm can be divided into three operational methods: DRC1, DRC2, and DRC3. The methods DRC1, DRC2, and DRC3 predict the user location using DR, and then they update the position through measurements obtained from WiFi/iBeacon RSS values and pedestrian direction data using NBC-based fingerprinting algorithm. For the positioning accuracy comparison between DSPF and other filter methods including PF, UKF, and KF in our experimental tests, other filters are also executed by DR, DRC1, DRC2, and DRC3. That is, in a manner similar to the four operational modes of the DSPF-based method as shown in Table 2, the positioning methods based on the PF, UKF, and KF predict the user position using the user motion information obtained from the IMU sensors (i.e., DR), and they correct the predicted position through positional measurements gained by the user direction information and WiFi/iBeacon RF signals using the NBC-based fingerprinting method (i.e., DRC1, DRC2, and DRC3).

**Figure 6:**
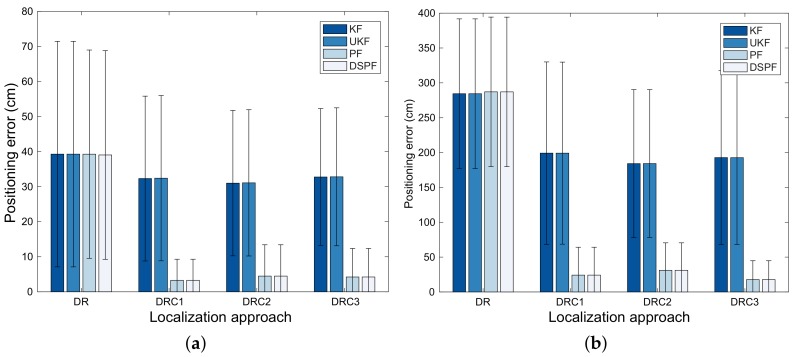
Positioning error of the localization schemes for test cases (**a**) TC1 and (**b**) TC2.

For test cases TC1 and TC2, the means and standard deviations for the positioning error obtained by the Bayes filters from 50 pedestrians are represented by Figure 6. This figure is illustrated by bar charts with error bars that consist of thin vertical lines and short horizontal line segments to show the reliability or precision in a set of position estimation values obtained from Bayes filters. The error bars are added to the bar charts based on the standard deviation for the positioning error obtained by the filters. Comparing results from DR, DRC1, DRC2, and DRC3 in Figure 6 reveals that the filters that employs both the DR and position correction can provide more accurate and reliable positioning results than those using only DR. Particularly, for TC2 with the bad radio environment, when DRC1, DRC2, and DRC3 are applied, the localization error for the filters is decreased remarkably. From Figure 6, we can also see that the performances of DRC1, DRC2, and DRC3 are not influenced largely by types of RSS values employed to obtain the observations of the user position. For all positioning algorithms in test cases TC1 and TC2, DSPF achieves lower positioning error compared with UKF and KF, while providing localization results that are as accurate as PF, as shown in Figure 6. Moreover, it is shown in Figure 6 that since DSPF accomplishes a smaller standard deviation for the localization error compared to Kalman filtering-based methods and provides comparable positioning error standard deviation to PF, it can carry out the position estimation that is as reliable as PF.

**Figure 7:**
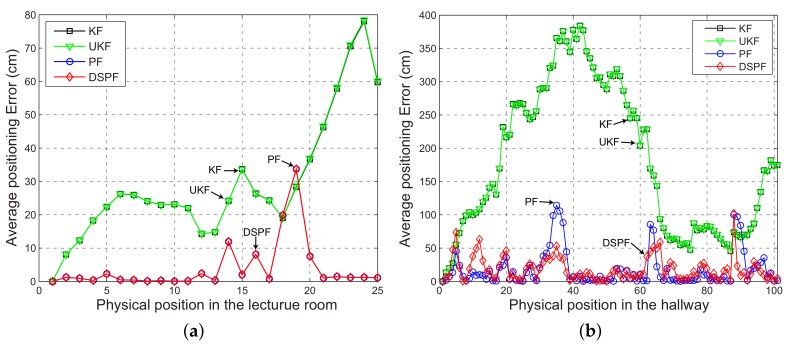
Mean positioning error for the localization scheme in which the Bayes filters are performed by DRC3 at each physical location (orange circle and green square in Figure 4) of test cases (**a**) TC1 and (**b**) TC2.

**Figure 8:**
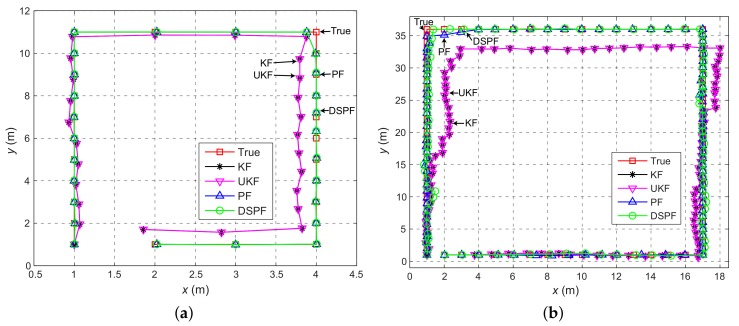
Pedestrian trajectories calculated by the positioning scheme in which the Bayes filters are performed by DRC3 when striding on the physical positions (orange circles and green squares in Figure 4) of test cases (**a**) TC1 and (**b**) TC2 clockwise. The trajectories are indicated with *x* (east) and *y* (north) coordinates.

The positioning error can be analyzed in more detail by observing Figure 7 and Figure 8, which represent the average value of the positioning error and the user’s trajectory estimated by the localization scheme in which the Bayes filters are executed by DRC3 in test cases TC1 and TC2. For TC1, the positioning accuracy difference between DSPF and Kalman filtering-based methods is remarkable. Even for TC2 with the bad wireless conditions, the localization results of DSPF against UKF and KF are greatly enhanced, especially around the location numbers ranging from 20 to 60 with low values of packet success rate, as shown in Figure 5. This test results suggest that the localization system based on DSPF can offer reliable and accurate positions of the user even in the case where there are many obstructions or in bad wireless condition. This is probably because the DSPF algorithm can execute the position estimation adaptable to the change in indoor wireless environments by using the target distribution that reflects indoor wireless environments surrounding the user through the multiple observations, as described in Section 3.4.

### 5.2. Effect of Sample Size

**Figure 9:**
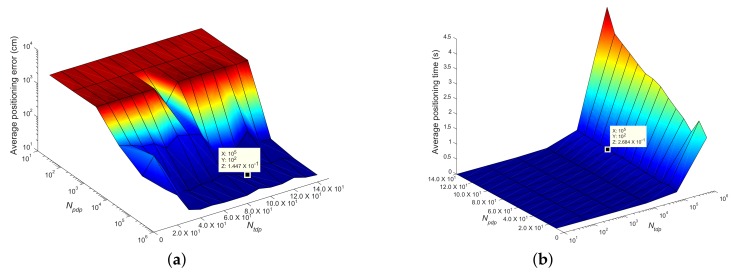
Positioning experiment results of test cases TC1 and TC2 executed by positioning scheme DRC3 using DSPF for the values between $N_{pdp}\subset \left\{10,140\right\}$ and $N_{tdp}\subset \left\{10^{1},10^{6}\right\}$; (**a**) Curve of the average positioning error and (**b**) Curve of the average positioning time. Text annotations on the curves indicate optimal values of $N_{pdp}=10^{2}$ and $N_{tdp}=10^{5}$ that can achieve a high degree of positioning accuracy at little computational cost in TC1 and TC2.

This subsection explores the effect of the number of the proposal distribution particles $N_{pdp}$ and the number of the target distribution particles $N_{tdp}$ of DSPF on the accuracy and execution time of our localization algorithm. Our experiments were executed to determine the optimal $N_{pdp}$ and $N_{tdp}$ needed to estimate the pedestrian position accurately and rapidly in test cases TC1 and TC2. In the experiments, the positioning accuracy and execution time were obtained from 30 runs of the positioning algorithm performed by DRC3 based on DSPF. They were calculated according to $N_{pdp}$ and $N_{tdp}$ to emphasize the effect of the change in $N_{pdp}$ and $N_{tdp}$ and to verify their optimal values, as represented in Figure 9a,b.

The optimal values of $N_{pdp}$ and $N_{tdp}$ are decided based on the curves of the average positioning error and average execution time obtained from the experiments of TC1 and TC2. As can be noticed in Figure 9a,b, as $N_{pdp}$ and $N_{tdp}$ decrease, while the computational time for the position estimate becomes lower, the positioning error increases. The increase in the localization error is caused by a lack of diversity among the particles used to represent the position where the pedestrian is likely to be located. On the contrary, the selection of a high value of $N_{pdp}$ and $N_{tdp}$ increases the positioning time, but it does not necessarily lead to a higher positioning accuracy. They are finally fixed to $N_{pdp}=10^{2}$ and $N_{tdp}=10^{5}$. These values allow a minimum value of the localization error with less positioning time in TC1 and TC2. The choice of $N_{pdp}$ and $N_{tdp}$ always entails a risk, which can be understood as a tradeoff between positioning accuracy and execution time.

### 5.3. Computation Cost

**Figure 10:**
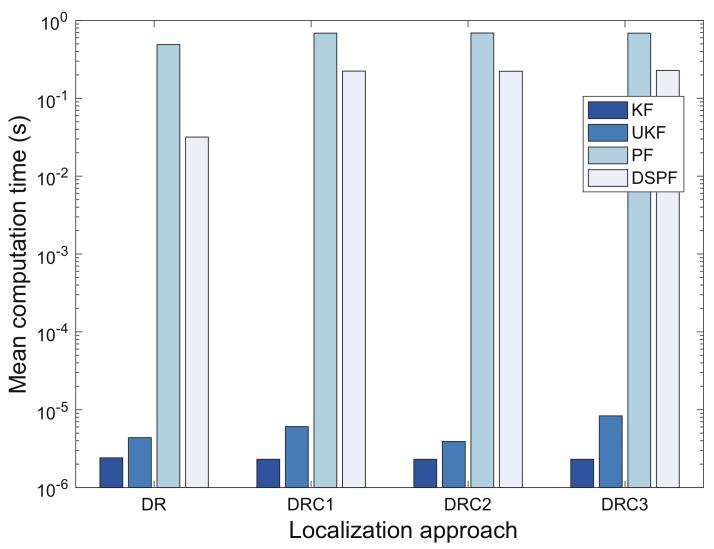
Mean computation time of localization approaches.

Figure 10 shows the mean computation time of the localization schemes for test cases TC1 and TC2. The PF that requires many particles to estimate the model state can result in huge computational cost. Its computational complexity is $O(N_{s})$, where $N_{s}$ is the number of the particles [48]. The DSPF algorithm requires $O(N_{pdp})$ operations that locate the user in every time step.

The resampling algorithm is essential for guaranteeing better estimation performance of the PF, but can be very computationally costly. For this reason, various resampling schemes for particle filters suitable for real-time implementation have been proposed, including resampling algorithms with reduced computational complexity and parallelization of resampling with multicore processors [49,50]. This study does not aim to design a new resampling scheme for the PF. The DSPF and PF for our experiments used only the systematic method [46] for resampling, which was applied to $N_{s}$ particles for PF and $N_{pdp}$ samples of the proposal distribution for the DSPF. For this experiment, the DSPF algorithm could carry out the position estimation with less particles for the proposal distribution than sample size of PF by using the target distribution that reflects indoor wireless environments surrounding the user; that is, PF used $10^{3}$ particles based on the settings of the values in some sensitivity experiments [26], and $N_{pdp}$ and $N_{tdp}$ used in DSPF were set to $10^{2}$ and $10^{5}$ by analyzing the empirical data respectively, as addressed in Section 5.2. For this reason, the DSPF algorithm for our experiments could reduce the number of operations required for the resampling as well as weight computation using the target distribution. As a result, the DSPF required a lower computational cost than PF to infer the pedestrian location in positioning system; therefore, DSPF in DR, DRC1, DRC2, and DRC3 could conduct 93.51%, 67.48%, 67.79%, and 66.83% faster positioning compared with PF, respectively. However, different choices of $N_{pdp}$ and $N_{tdp}$ may be needed for more complex filtering problems, especially when the DSPF is applied for the high-dimensional nonlinear systems.

When the dimensions for the state $x_{k}$ and measurement $z_{k}$ are $L_{x}$ and $L_{z}$ respectively (i.e., $x_{k}\in R^{L_{x}}$ and $z_{k}\in R^{L_{z}}$), the computation time complexities for UKF and KF are generally $O(L_{x}^{3})$ and $O(L_{z})$ respectively [51,52]. In our experiments, because $L_{x}$ is equivalent to $L_{z}$, UKF requires a more computational time than KF, as shown in Figure 10. Furthermore, DSPF and PF have a higher computational cost than UKF and KF, owing to the use of many samples (particles).

## 6. Conclusions

Most indoor pedestrian positioning methods are affected by considerable errors owing to the RF signals that can vary over space and time, the user’s movements, and the use of cheap MEMS devices (gyroscope and accelerometer). To solve this issue, this paper introduced a positioning system that employs a machine learning-based fingerprinting approach that leverages the user’s direction information and RSS value as training data, DR, and improved particle filter called DSPF. The DR approach predicts user locations using the motion information (direction and acceleration) measured from the IMU sensors on a mobile phone. For our localization system, the measurements for the user location are determined by the fingerprinting method without GNSS indoors. The user position data gained by the fingerprinting or DR method can have large errors. The DSPF algorithm in the indoor positioning scheme is employed to achieve more accurate localization results by combining noisy location information gained by the fingerprinting and DR. The DSPF algorithm based on particle representations with the target and proposal distributions can conduct the position estimation adaptable to the change in indoor wireless environments by using the target distribution that reflects indoor wireless environments surrounding the user through multiple observations. Experimental results denote that the DSPF algorithm in the positioning system can provide more accurate positioning results compared with Kalman filtering-based methods, accomplishing a position estimation that is as accurate as PF while providing higher computational efficiency than PF. We aim examine the positioning performance of the DSPF for pedestrians that movie from place to place in more complex indoor environments in the near future, including airports (large open spaces), shopping malls, and libraries.

## Figures and Tables

**Table 1:** Summary of indoor positioning approaches associated with the positioning algorithm suggested in this paper.

Method	Technique	Environment	Sample	Accuracy Mean
			Size	Error (m)
Evennou and Marx [22]	DR, WiFi RSS	Corridor and room in	10,000	1.53 m mean
	fingerprinting, KF,	indoor office building		
	and PF	(40 m × 40 m)		
Nurminen et al. [23]	DR, WiFi RSS	Corridor in	400	2.0 m mean
	fingerprinting, PF,	campus building		
	and smoother	(95 m × 61 m)		
Xie et al. [24]	DR, magnetic		5000	2.0 m mean
	fingerprinting, and	Hall, conference room,		
	reliability-augmented PF	corridor, and library		
GIFT [25]	DR, RSS gradient-based	Five-story	2000	5.6 m in 80%
	fingerprinting, and	office building		
	extended PF	(8000 $m^{2}$)		
SLAC [11]	DR, WiFi RSS		60	6 m medianin airport and 3.8 mmedian in atrium
	fingerprinting, convex	Airport (10,000 $m^{2}$) and		
	optimization localization,	campus atrium (4000 $m^{2}$)		
	and specialized PF			
Sung et al. [26]	DR, WiFi/iBeacon	Corridor and room in	4	0.7 m mean
	RSS fingerprinting,	campus building		
	and SKPF	(37.3 m × 26.5 m)		
Proposed Scheme	DR, WiFi/iBeacon	Corridor and room in	100	0.3 m mean
	RSS fingerprinting,	campus building		
	and DSPF	(37.3 m × 26.5 m)		

**Table 2:** Localization Schemes for Experimental Tests.

Notation	Description
DR	Dead reckoning (position prediction) via heading angles and accelerations
DRC1	DR and position correction (update) via observation determined by heading and WiFi RSS data
DRC2	As in DRC1, but for observation determined by heading and iBeacon RSS data
DRC3	As in DRC1, but for observation determined by heading, WiFi RSS, and iBeacon RSS data
